# First Results of Field Absolute Calibration of the GPS Receiver Antenna at Wuhan University

**DOI:** 10.3390/s151128717

**Published:** 2015-11-13

**Authors:** Zhigang Hu, Qile Zhao, Guo Chen, Guangxing Wang, Zhiqiang Dai, Tao Li

**Affiliations:** 1 GNSS Research Center, Wuhan University, Luoyu Road No. 129, Wuhan 430079, China; E-Mails: zhigang.hu@whu.edu.cn (Z.H.); gxwang@whu.edu.cn (G.W.); dzq@whu.edu.cn (Z.D.); taoli@whu.edu.cn (T.L.); 2 School of Geodesy and Geomatics, Wuhan University, Luoyu Road No. 129, Wuhan 430079, China

**Keywords:** GPS, IGS, receiver antenna, field absolute calibration, absolute antenna phase center corrections

## Abstract

GNSS receiver antenna phase center variations (PCVs), which arise from the non-spherical phase response of GNSS signals have to be well corrected for high-precision GNSS applications. Without using a precise antenna phase center correction (PCC) model, the estimated position of a station monument will lead to a bias of up to several centimeters. The Chinese large-scale research project “Crustal Movement Observation Network of China” (CMONOC), which requires high-precision positions in a comprehensive GPS observational network motived establishment of a set of absolute field calibrations of the GPS receiver antenna located at Wuhan University. In this paper the calibration facilities are firstly introduced and then the multipath elimination and PCV estimation strategies currently used are elaborated. The validation of estimated PCV values of test antenna are finally conducted, compared with the International GNSS Service (IGS) type values. Examples of TRM57971.00 NONE antenna calibrations from our calibration facility demonstrate that the derived PCVs and IGS type mean values agree at the 1 mm level.

## 1. Introduction

**Figure 1:**
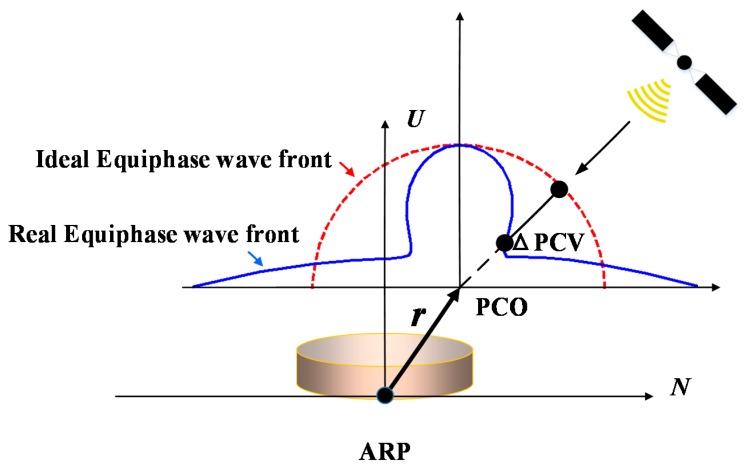
Antenna phase center description.

As it is well known, carrier phase observations, which undoubtedly play a dominant role in GNSS high precision applications, are generated between the antenna electrical phase centers of satellite and receiver. Ideally, the receiver antenna would act as a spherical phase response for GNSS signals [1]. However this is not true in most of cases due to the fact that the antenna electrical phase center is neither a physical or mechanical point that can be reachable by a real measurement tool, nor a unique well-defined one in the whole signal reception range [1,2]. Instead, the electromagnetic behavior of antennas is not homogeneous and the location of its phase center varies with different elevation and azimuth directions of the received signals [1,2,3,4,5]. The difference between the distorted real wave front and the perfect one causes phase measuring errors. The magnitudes of these unexpected phase measure errors (dependent on different receiver types) vary from a few millimeters up to several centimeters [3,6,7,8], which becomes one of the largest limiting factors to obtain cm- even mm-level high-precision positioning results [1,9]. The antenna electrical phase center can be defined as the absolute mean phase center offset (PCO) with respect to the antenna reference point (ARP) and the elevation and azimuth dependent phase center variations (PCVs) [3] (Figure 1). Neglecting antenna PCO and PCV correction will therefore lead to a significant position bias, especially in the height component (cm-level error), and result in an unreasonably large terrestrial scale bias in global GPS solutions with respect to results from Very Long Baseline Interferometry (VLBI) and Satellite Laser Ranging (SLR) [3,9,10]. Furthermore, the tropospheric propagation delay error is not easily separated from the antenna phase center bias. Both of them vary with different elevations, and result in height biases when tropospheric zenith delays are estimated. These problems have been confirmed by many research groups, see [2,3,7,11,12].

Many international research groups in the last nearly two decades have succeeded in exploring several antenna phase center calibration approaches so as to solve the problems discussed above. The relative antenna PCO and PCV are firstly defined in field procedures based on a very short baseline with one reference antenna at one end and the one to be calibrated on the other end [2,3]. Although many antenna calibrations are performed relative to the same one, the relative calibration results reflect somewhat station-specific errors, e.g., multipath errors. Then a calibration procedure in an “absolute” sense has been implemented to separate the antenna to be calibrated PCO and PCV independently of the reference antenna [4,5,13]. There is one thing that should be pointed out that if the reference antenna has been calibrated in another way (e.g., results from different calibration institutes or anechoic chambers), then the relative antenna PCO and PCV can be transited to absolute ones [14,15]. Nevertheless, high precision anechoic chamber calibration results have demonstrated that the absolute field calibration using GNSS data can reach a good agreement at the 1-mm level with each other [1]. Absolute PCCs are required for state-of-the-art GNSS cm- or mm- level positioning applications to avoid the possible scale problem in the coordinate solutions and systematic station errors (especially in the height component), e.g., regional and worldwide permanent reference station networks, equipped with mixed receiver and antenna types.

In November 2006, the elevation and azimuth-dependent PCC models, most of which derived from well-designed robot calibration systems, were recommended by IGS (IGSMAIL-5438 by Gendt, 2006). Although there may exist different production series for one antenna type used in precise geodetic measurements, it was generally assumed that one geodetic antenna type has a quite stable and similar phase pattern. However, this assumption is not always true for all the GNSS antenna types. As a rebuttal to this assumption, Wübbena *et al.* [8] found that in some cases, the phase patterns between two antennas of the same type have a large difference amounting to several millimeters, which was further demonstrated by using a precise point positioning (PPP) analysis [16] and precise relative positioning experiments based on a very short baseline [17]. This indicates that individual absolute antenna calibrations, rather than using IGS type mean values, are recommended if tasks related to GNSS precise positioning are conducted.

China has launched a large research project, namely, “Crustal Movement Observation Network of China” (CMONOC), which is a comprehensive observational network for real-time dynamic monitoring of changes in continental tectonic settings and to explore their effect on resources, the environment and disasters. The main observation technique in this project is the well-known GPS, combined with other space-based techniques, such as VLBI and SLR [18]. As the core observation technique within the CMONOC project, the GPS permanent networks consist of more than 200 tracking stations, which were equipped with different receivers and mixed antenna types, and some of them without precise calibration for GNSS antenna phase center. Aimed at high precise dynamic crustal movement monitoring, it is still necessary to carry out individual absolute antenna recalibrations. Therefore, one of goals of this project was to establish an absolute field antenna phase calibration platform using GPS to remove or greatly reduce the detrimental effect of phase center errors on mm-level precise geodetic and geophysical applications. The absolute field antenna phase calibration platform was established on the rooftop of a laboratory building on the campus of Wuhan University (China). Based on this calibration platform, a direct absolute calibration in field procedure has been attempted in this paper.

## 2. The Absolute Antenna Phase Calibration Platform at Wuhan University

**Figure 2:**
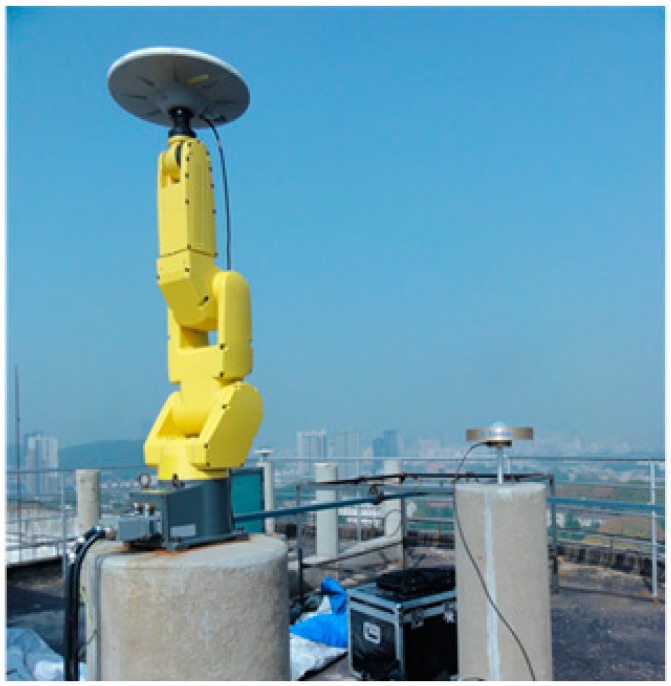
Robot antenna calibration facility at Wuhan University.

To precisely calibrate any test antenna, a calibration system has been strictly established on the rooftop of a laboratory building at Wuhan University, see Figure 2. This calibration platform is composed of two solid concrete pillars with a height of 1.6 m above the ground for the purpose of reducing multipath signals reflected from the ground. These two pillars lie along a south-north line separated by approximately 3 m. A very short baseline is necessary for high-precise antenna calibration to remove the satellite orbit errors, tropospheric or ionospheric propagation path delays and partial multipath effects in the differential model [2]. An arbitrary antenna is placed on one end of the baseline as a base station, and a flexible robot instrument with an antenna mounting point was set up on the other end. The accuracy of test antenna calibration certainly benefits from high quality phase observations. Therefore, both the test and the reference antenna are connected to a set of Trimble Net R9 geodetic GNSS receivers, respectively. It should be mentioned that in our procedure it is unnecessary to know the reference antenna PCO and PCV values.

## 3. Algorithm and Strategies of Data Processing

### 3.1. Antenna Phase Model Review

There may exist different antenna definitions to describe the inhomogeneous electromagnetic behavior of antennas in different application aspects. However, an antenna definition consists of a mean antenna offset (PCO) with respect to an antenna reference point (ARP) and azimuth and elevation dependent phase center variations (PCVs) are commonly adopted in the GNSS community [2,3,4]. The ARP has been conventionally defined as the intersection of the vertical antenna axis of symmetry with the bottom of the antenna.

A set of complete antenna phase center models is usually defined in a left-handed local coordinate system. The reference origin is from the ARP. The Up direction parallels the vertical antenna symmetric axis and points to the zenith direction. The north direction actually aligns with the geographic north direction in field measurements within the horizontal plane. The so-called signal frequency dependent 3D mean phase center offset (PCO) is referring to the reference origin (ARP), and finally the elevation and azimuth dependent PCVs are therefore referring to the PCO for individual frequencies. Then the total PCCs in any direction of a satellite can be given by:

(1) ∆r=r ⋅ u+PCV(α,β)

where *r* and *u* are the 3D PCO vector and the unit vector in the satellite sight, respectively; *α* and *β* are the azimuth and elevation of satellite in the antenna local coordinate system (*cf.* Figure 1). In Equation (1), the PCO impacts are dependent on the elevation mask [6], and the magnitude of the azimuth dependent PCVs are generally small and are neglected directly in the relative antenna PCCs in differential model [2]. However, the azimuth dependent PCV values of some antenna types can reach up to 1–2 cm due to the unsymmetrical PCV patterns of the antennas [8]. This should not be ignored in this situation if highly accurate positions are required.

### 3.2. Multipath Analysis

**Figure 3:**
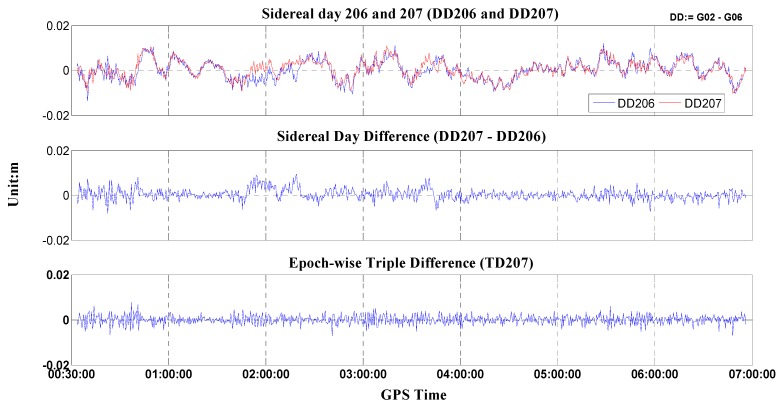
Multipath migration using sidereal day differences and epoch-wise TD.

GNSS phase multipath is a main error source in absolute and relative determination of antenna phase center variations due to the fact that there hardly exists an environment unaffected by multipath effects [8]. In order to derive precise PCV-independent stations in field procedures, multipath must be separated carefully from PCVs in the data processing [5], but it is impossible to exploit a perfect mathematical model to precisely describe multipath which is suitable for any general station situation. However, MP can be considered as time-variant error source since it fluctuates with time in the local station environment. Instead, PCVs can be treated as static parameters which are only dependent on elevation and azimuth (*i.e.*, antenna type dependent). Therefore, the MP and PCV are essentially independent from each other and can be separated with different measurements and strategies. In this paper, three measures were taken to mitigate multipath as much as possible: (1) choosing a less multipath-disturbing field. The calibration baseline is situated on top of the roof of a teaching building, with no significant sky obstructions and without nearby electromagnetic interference. The two solid concretes were intentionally built at 1.6 m high up the ground to effectively reduce multipath sources from ground reflections; (2) Double observables differencing with two stations. There is no doubt that two stations at both ends of a short baseline are always under similar multipath conditions. The station distance in our calibration procedure is about 3 m. Double differencing (DD) with these two stations will differentially eliminate most parts of any multipath effect; (3) Time-wise triple difference. The remaining multipath of DD, which will still disturb antenna phase pattern modeling (see Figure 3, demonstration only), cannot be neglected. One can triple difference the DD observables of two successive mean sidereal days since multipath periodically appears after a mean sidereal day [4]. Although the so-called sidereal filter approach is able to further reduce multipath, it takes several continuous days to accomplish an antenna calibration. In addition, partially differential multipaths may not work for some signals from day to day due to the flaky weather, e.g., 2:00 GPST to 4:00 GPST in the Figure 3. In our calibration approach, similar to [8,19], we adopt the epoch-difference approach which is more efficient due to the fact that a change of the multipath behavior in a very short time is extremely similar. The epoch-differences on the basis of DD observations results demonstrate that the multipath has further reduced (*cf.* Figure 3).

### 3.3. Absolute PCO and PCV Separated from Relative Observation

**Figure 4:**
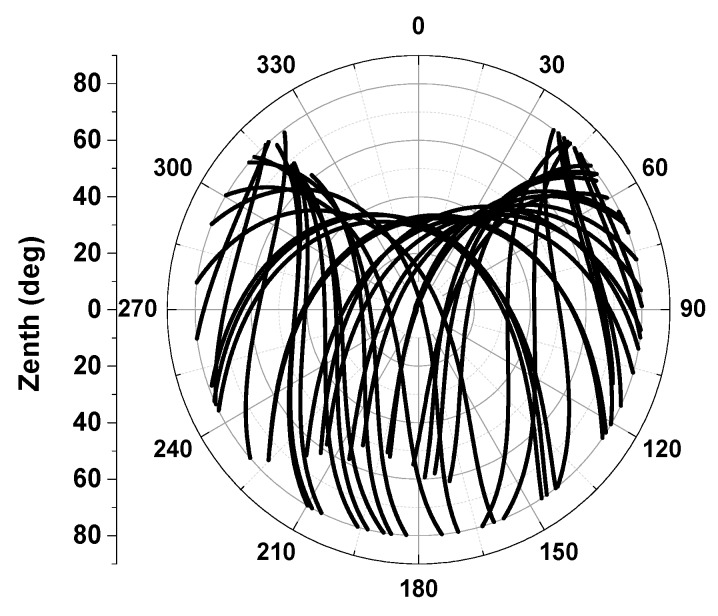
Observations coverage on antenna hemisphere when the antenna was fixed (24 h static, DoY2014207 in Wuhan).

Forming a short baseline with the test antenna and an arbitrary antenna is required for the sake of removing most common errors, e.g., satellite orbit and clock errors, ionospheric and tropospheric propagation path delay errors, *etc.* The relative observables also verify that multipath is greatly reduced, however, this may lead the antenna phase center depending on the antenna placed on the base station (see Figure 2). To access the test antenna PCO and PCV in an absolute way, one can subtract the same baseline scenario’s observables of two successive mean sidereal days with a rotation of the test antenna in the horizontal orientation on the second day [4]. Without a change of rotation, the phase center variations will also be eliminated when double differences are formed. Furthermore, tilting w.r.t a horizontal axis is also needed to avoid possible station-dependent “Northern Hole” effects, see Figure 4 Observation coverage on the antenna hemisphere when the antenna was fixed (24 h static, DoY2014207 in Wuhan). To meet the requirement of a large range of motions (rapidly rotating and tilting), a precise 6° of freedom robot (nominal repeatability precision better than 0.1 mm) is introduced in our calibration procedure as to improve calibration accuracy and sharply shorten the calibration running time.

The complete data processing for GPS antenna calibration used in this study consists of three steps. The collected GPS data are firstly used to form well known double difference (DD) observation equations. After the DD ambiguities are fixed, then the triple difference (TD) between two successive epochs are performed for step of absolute PCOs derivation, followed by PCVs estimation derived from TD observation residuals. Our data processing procedure is different from that used in [5] for absolute antenna calibrations. While they derive an absolute test antenna’s PCO and PCVs by forming the time difference of a single difference (TDSD) observable (one satellite only), this kind of calibration requires a common clock at the reference and test antennas, though it is well handled in the whole procedure. In addition, the TDSD model removes the ambiguities without taking ambiguity integer characteristics into account. In our procedure, the TDSD observations are further formed DD observations between two satellites which do not only remove receivers’ clock bias, but also easily fix the DD ambiguity using so-called LAMBDA [20] based on integer least squares (ILS). As fixing correct ambiguities is a priority to achieving the highest accuracies and reliability of PCCs, we validate the estimated ambiguities using Ps-LAMBDA [21] based on ambiguity success rates.

Before the start of the calibration procedure, several known factors must be mentioned: (1) The baseline between the reference and test XYZ monument positions were well-determined using GPS single frequency solutions on a short baseline; (2) Coordinate transformation. Although the repeatability precision of the introduced robot is announced to be better than 0.1 mm, the robot output Cartesian coordinate values is w.r.t robot local coordinate system, which is different from the GNSS one (e.g., WGS-84 in this paper) Therefore, a seven-parameter transformation program has been implemented to resolve the coordinate transformation parameters between the robot local coordinate system and WGS-84; (3) Time synchronization. Every visible satellite’s elevation and azimuth w.r.t the antenna-fixed coordinate system (was set to parallel with robot local coordinate system) has to be computed in the course of the robot’s rotating and tilting. Therefore, it is quite clear that those motions must be directly connected to GPS time for synchronization with the phase observations. Unfortunately, the time synchronization function in the control robot system is not available at the moment. We actually used a Personal Computer (PC) to record the robot’s moving time and sets of spatial coordinates and attitudes w.r.t the robot local orthonormal coordinate system and further implemented a straight-forward approach of time synchronization to correct the used PC clock (synchronization accuracy better than 0.1 s) in an hourly fashion using the GPS GGA time tag output from one of GPS receivers in our calibration facility.

After coordinate transformation and time synchronization, the GPS observables and robot motion data can be easily merged together, *i.e.*, any vector *a* in robot local orthonormal coordinate system can be transformed to *b* in the GPS WGS-84 coordinate system using well-known seven parameter Helmet transformation model, read as:

(2) $$b=x_{0}+\left(1+m\right)R\left(\alpha ,\beta ,\gamma \right)a$$

where, *x*_0_ is coordinate shift components, *m* is a scale factor (*m* = 0 in this paper), and *α*, *β*, *γ* are the Eulerian angles. *R* is rotation matrix from robot local coordinate system to GPS WGS-84 coordinate system.

For a very short baseline, the linearized phase double difference (DD) observation equations in meters from the location of ARP between reference antenna and the antenna under test can be simply read as:

(3) $$\nabla ∆\emptyset_{1,2}^{i,j}=-∆a\;\cdot \;r_{1}+∆b\;\cdot \;r_{2}-∆{PCV}_{1}^{i,j}\left(az,el\right)+∆{PCV}_{2}^{i,j}\left(az,el\right)+\nabla ∆{MP}_{1,2}^{i,j}+\nabla ∆N_{1,2}^{i,j}+\nabla ∆\varepsilon_{1,2}^{i,j}$$

The subscripts 1, 2 stand for reference and test receiver antennas and *i*, *j* for satellites. Notations of *∇*∆*N*, *∇*∆*PCV* and *∇*∆*ε* stand for DD ambiguities, DD phase center variations and some expected random phase noise for real observations, respectively. *az*, *el* are azimuth and elevation w.r.t antenna ARP. ∆*a* and ∆*b* are the SD linearized coefficients of line of sight between receiver and satellite. Vectors *r*_1_ and *r*_2_ stand for the PCO from the location of ARP for reference antenna and test antenna. The coordinate vector between locations of ARP of reference antenna and test antenna at any epoch are well-determined with high precise robot motion data and coordinate transformation parameters (see Equation (2)) and this geometry quantity is removed when Equation (3) is linearized. Additionally, the atmospheric propagation delay error (ionosphere and troposphere) and both of receiver and satellite clock biases are considered equivalent for each antenna and these common errors are eliminated since the distance of baseline in our procedure is as short as about 3 m. However, the multipath term *∇*∆*MP* errors may be on the order of a few millimeters in terms of phase path, and therefore cannot be neglected in the DD linearization.

Intuitively, the expected test antenna absolute PCO and PCV cannot be extracted if the reference antenna PCO and PCV are not available in Equation (3). Nevertheless, an absolute antenna calibration can be accessible if the reference antenna phase center is properly eliminated or greatly reduced and it does not move during the calibration procedure. This can be achieved by rotating and tilting the test antenna mounted on the end of a robot (see Figure 2). The time difference (also Triple difference, TD) of successive two set of antenna orientations in a short time, generally several seconds, can separate the test antenna PCO and PCV from DD observables (see Equation (3)). We expand Equation (3) for times *t_A_* and *t_B_*, and ignore superscript satellite symbol *i* and *j*, reads:

(4) $$\begin{array}{ll} \nabla ∆\emptyset_{1,2}^{t_{B}}-\nabla ∆\emptyset_{1,2}^{t_{A}} & =∆b^{t_{B}}r_{2}-∆b^{t_{A}}r_{2}-∆a^{t_{B}}r_{1}+\nabla ∆a^{t_{A}}r_{1}-∆{PCV}_{1}\left({az}^{t_{B}},{el}^{t_{B}}\right)\\ & +∆{PCV}_{1}\left({az}^{t_{A}},{el}^{t_{A}}\right)+∆{PCV}_{2}\left({az}^{t_{B}},{el}^{t_{B}}\right)-\nabla ∆{PCV}_{2}\left({az}^{t_{A}},{el}^{t_{A}}\right)+\nabla ∆\varepsilon_{1,2}^{t_{B}}\\ & -\nabla ∆\varepsilon_{1,2}^{t_{A}} \end{array}$$

Note that, the DD ambiguities in Equation (3) can be easily fixed, e.g., using well known LAMBDA [20], and be eliminated as constant values in Equation (4). As discussed above, the multipath errors were verified to be sharply reduced when the time difference of DD is performed. Therefore the $\nabla ∆{MP}_{1,2}^{t_{B}}-\nabla ∆{MP}_{1,2}^{t_{A}}$ term has also been removed from Equation (5). In a very short time interval $t_{B}-t_{A}$, a slight change of the elevation and azimuth from static reference station to satellite leads to a negligible PCO and PCV increment, $∆a^{t_{B}}r_{1}-∆a^{t_{A}}r_{1}+∆{PCV}_{1}\left({az}^{t_{B}},{el}^{t_{B}}\right)-∆{PCV}_{1}\left({az}^{t_{A}},{el}^{t_{A}}\right)<1\;\text{mm}$, in Equation (4). On the contrary, the PCO and PCV of the test antenna with the robot help of rapidly moving through a large range of motions, *i.e.*, $z_{test}^{t_{A}}\neq {az}_{test}^{t_{B}}$, ${el}_{test}^{t_{A}}\neq {el}_{test}^{t_{B}}$, cannot be disregarded directly and still remain in Equation (4). Now, taking these factors [20] into account, Equation (4) can be simply rewritten as:

(5) $$\nabla ∆\emptyset_{1,2}^{t_{B}}-\nabla ∆\emptyset_{1,2}^{t_{A}}=\left(∆b^{t_{B}}-∆b^{t_{A}}\right)r_{test}+∆PCV_{test}\left(az^{t_{B}},el^{t_{B}}\right)-∆PCV_{test}\left(az^{t_{A}},el^{t_{A}}\right)+\nabla ∆\varepsilon_{1,2}^{t_{B}}-\nabla ∆\varepsilon_{1,2}^{t_{A}}$$

After the short time difference of consecutive epochs, the PCO and PCV terms of the reference antenna are safely removed. Finally, the PCO and PCV of the test antenna are successfully transferred from the sense of “relative” to “absolute”. For a convenient implementation, the PCO of the antenna is firstly estimated in a least squares sense without PCVs, and then when PCO has been adjusted and removed as constant in Equation (5), the PCVs of the test antenna are therefore accessed using a lower degree and orders of surface spherical harmonics to fit the TD residuals in Equation (5).

## 4. Data Collection and Result Discussion

### 4.1. Experiment Data Collection

In order to achieve 1 mm level absolute antenna phase center calibration it is necessary that all possible orientations should be covered with highly dense and homogeneous observations, so that station dependencies such as blank holes can be avoided. Meanwhile, observations down to zero elevation are recommended. However, it is simply not possible to satisfy this requirement in a static relative calibration procedure, even if tracking for as long as 24 h (see Figure 4).

**Figure 5:**
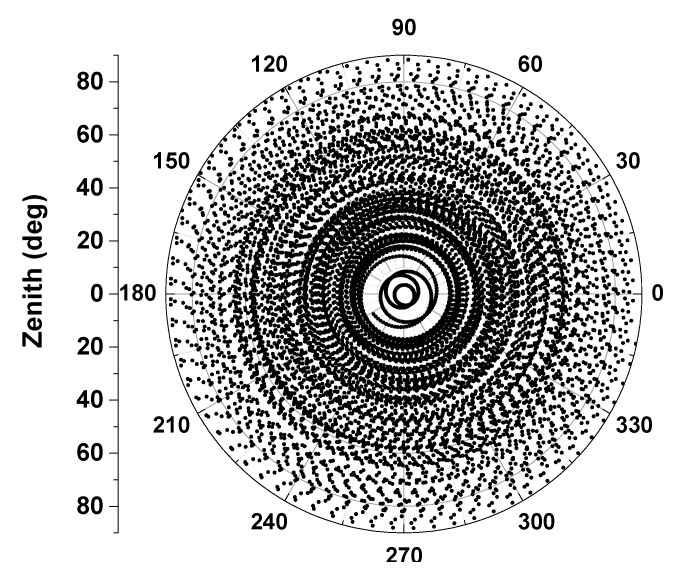
Observations coverage on test antenna hemisphere when the antenna was being rotated (4 h rotating and tilting mounted on the robot, DoY2014240 in Wuhan).

To avert such defects, a high-precision robot which can be flexibly rotated and tilted was introduced in our calibration procedure. It takes only several hours to collect data from several thousand precise positions (required for high precise derived PCO and PCV) and angle changes. This automatic observation procedure will stop when the full coverage of the test antenna pan is reached (see Figure 5).

Preliminary calibration experiments were performed to verify some key algorithms discussed in the paper and the implemented calibrating procedure. For the antenna phase center value validation, three types of GNSS geodetic antennas (TRM57971.00/NONE, TRM59800.00/NONE and LEIAR25/NONE) were chosen. These types of antenna have been calibrated by several institutes and published in the document igs05.atx and igs08.atx (ftp://igscb.jpl.nasa.gov/igscb/station/general/igs08.atx). Although some GPS-only solutions show that PCCs in igs08.atx, may cause small position offsets [16], the PCC model differences are very small: <0.5 mm, compared to igs05.atx. Therefore, the above three types’ PCCs in igs08.atx will be treated as true standards. In an absolute sense, the reference antenna can be arbitrarily chosen. However, a choke ring antenna Trimble TRM59800.00/NONE was used in the course of calibration for the sake of preserving observables from being affected by multipath errors (see Figure 2).

Our experiments were conducted on different days with the above three antenna types to investigate the absolute calibration repeatability precision based on the developed robot automation observation system. Among the experiments, the TRM57971.00/NONE type was calibrated during up to five sessions on different days and the other two types were tested in three sessions. Each experiment’s data collection was completed when the full coverage of the test antenna pan was reached (6–7 h on average with intervals of 1 s). Then the collected data sets were processed carefully (following Section 3.1 in this paper). Importantly, all the estimated ambiguities were fixed to integer values using LAMBDA and further investigated to make sure ambiguities were fixed with the highest possible ambiguity success rates [21,22] (lower bound >0.9999 for all the sessions).

### 4.2. Repeatability Accuracy Validation

**Figure 6:**
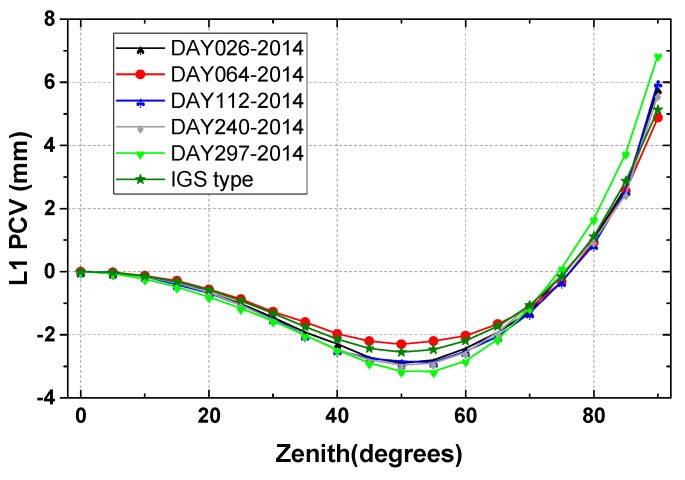
Zenith or elevation-only dependent PCVs of GPS L1 frequency, for the Trimble Zephyr Geodetic antenna TRM57971.00/NONE. The five days of independent calibrated results are in good accord with the IGS type for this antenna.

**Figure 7:**
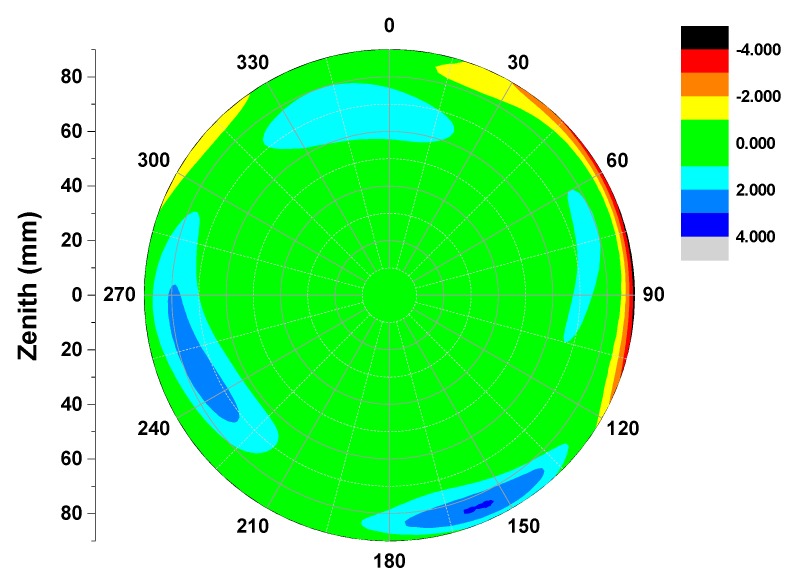
Differences of two independent calibration (DAY240 *vs.* DAY297-2014) derived full PCVs of GPS L1frequency (TRM57971.00). For the repeatability, some small gaps within 1 mm can be found in the mostly zenith range of 0–80°. Some parts of 2–4 mm discrepancies arose near the horizon of the antenna pan.

The repeatability accuracy can validate the stability and reliability of the calibrated antenna PCO and PCV values. We computed individual calibrations for the same antenna type TRM57971.00/NONE but from different days in 2014. The calibration results displayed in Table 1 show that there exist some slight discrepancies from each session, described by the standard deviations about 0.17 mm, 0.12 and 0.30 mm in the north-east-up components. Also the estimated PCOs apparently differ from each other in three components. The horizontal components (north and east) with sub-millimeter range, but the vertical component come up to almost 70 mm, which should not be neglected in high-precise positioning applications. Additionally, the elevation only and full PCVs (elevation and azimuth dependent) are also estimated for the test antenna (TRM57971.00/NONE), which are shown below Figure 6. Figure 6 demonstrates the agreements between five calibrated results and their discrepancies with IGS type values for the tested antenna. For the repeatability, some small gaps of ±0.5 mm can be observed in the zenith range of 0–80° calibrated on different days (gray line DAY240-2014 and green line DAY297-2014). These gaps increased at low elevation signal reception orientations, and an inconsistency of nearly 1 mm may be partly found (see Figure 6). The bad receiving behavior for most antennas at low elevations can be responsible for this [8], and this is true for full PCV estimation (see Figure 7). Furthermore, we expanded several additional calibrations to examine the performance of our calibrating procedure implemented in this paper. As shown in Table 1, both the test geodetic antennas TRM59800.00/NONE and LEIAR25/NONE show good repeatability accuracy. The standard deviations for the north, east and up components are better than 0.5 mm.

**Table 1:** PCO solutions for all sessions and the corresponding standard deviations.

Day of Year 2014	Antenna Type	North (mm)	East (mm)	Up (mm)	Standard Deviation (mm)
**026**	TRM57971.00/NONE	0.92	−0.10	66.92	*σ_N_* = 0.17, *σ_E_* = 0.12, *σ_U_* = 0.30
**064**		0.97	−0.25	67.01	
**112**		1.33	−0.38	66.68	
**240**		1.24	−0.35	66.99	
**297**		1.09	−0.34	67.50	
**026**	TRM59800.00/NONE	0.55	1.14	90.02	*σ_N_* = 0.04, *σ_E_* = 0.25, *σ_U_* = 0.26
**241**		0.61	1.49	89.66	
**065**	LEIAR25/NONE	1.33	0.78	155.77	*σ_N_* = 0.03, *σ_E_* = 0.10, *σ_U_* = 0.17
**113**		1.28	0.92	155.53	

### 4.3. Comparison with IGS Type Values

Besides the internal repeatability accuracy of the experiment itself, an external critical antenna calibration accuracy assessment with other calibration methods would be necessary, e.g., results from the anechoic chamber. However, due to the limitations of the experimental setup, the absolute antenna phase center calibrated results from the anechoic chamber are unavailable. Therefore, the published IGS antenna files like igs05.atx or igs08.atx contain most of common geodetic absolute antenna phase data sets calibrated by other international institutions or research groups. The test receiver antenna corrections in the igs08.atx stemmed from robot measurements by the well-known company Geo++. Referring to the calibration values from igs08.atx, we extended the assessments in Table 1 to Table 2. Assessments in Table 2 demonstrate that the calibrated values for the three types of antennas used in the experiments were estimated correctly and can be comparable, better than 0.5 mm, to those of IGS mean types in igs08.atx.

**Table 2:** PCO solutions accuracy compared to IGS mean types.

Antenna Type	*σ_N_* (mm)	*σ_E_* (mm)	*σ_U_* (mm)
TRM57971.00/NONE	0.16	0.1	0.23
TRM59800.00/NONE	0.03	0.17	0.18
LEIAR25/NONE	0.03	0.07	0.12

**Figure 8:**
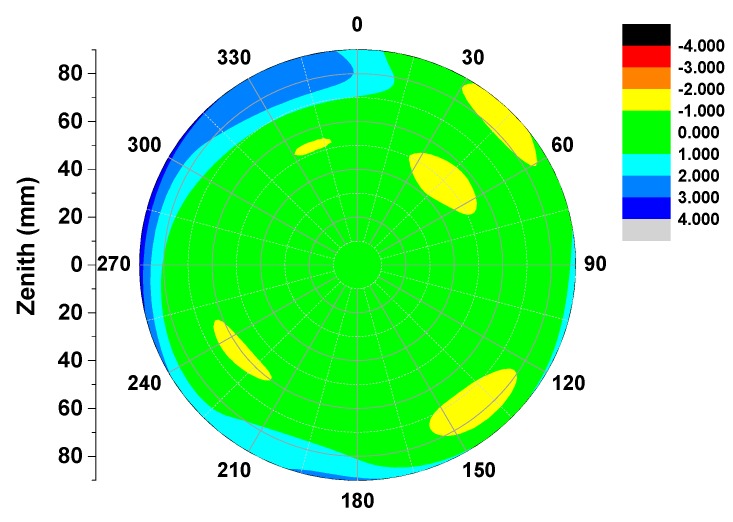
Differences of derived full PCVs (GPS L1 frequency) between the results of DAY3240-2014 and IGS standard values for TRM57971.00/NONE. An external consistency accuracy on the order of ±1 mm in the phase pattern can be achieved from zenith 75° to 0°, whereas the calibration accuracy at close to horizon typically fluctuated within 1–2 mm.

**Figure 9:**
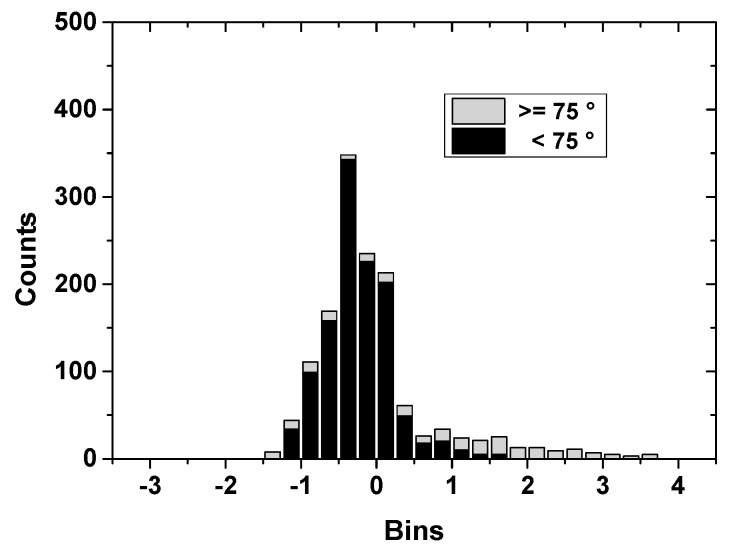
Frequency statistics on differences of derived PCVs from IGS type values, divided into two subsets (zenith angles below 75° (dark black) and above 75° (light gray)). Most of differences fell into ±1 mm bins and a small number went beyond 2 mm up to nearly 4 mm however.

Compared with the high accuracy calibration results in igs08.atx, the full derived PCVs in this paper show good consistency in the elevation range of 15–90° and ±1 mm absolute antenna phase center calibration accuracy can be safely obtained, see Figure 8 and Figure 9 (due to lack of space, only the example of DAY240-2014 is given). Some differences of 1–4 mm can be found close to horizontal reception angles below 15°, possibly caused by the remaining multipath effects and bad antenna receiving performance at low elevations in most of cases. Nevertheless, the consistence of two individual calibrations within the range of 1 mm above 15 elevations demonstrate the good performance of our calibration procedure and the calibration strategies for low elevation need to be further investigated in the future.

### 4.4. Conclusions and Future Work

After the absolute antenna phase center calibration algorithms were investigated, an absolute antenna phase center calibration procedure has been implemented and followed by a calibration platform based on a robot. Making use of the robot’s rotation and tilting, a homogenous observations distribution with regard to the antenna hemisphere are achievable, which is critical for high precision antenna phase center parameter estimation. The calibration results of GPS antennas presented in this paper show good repeatability for calibrations with different antenna types on different days. Our preliminary calibration results validate the absolute calibration methods of the robot and IGS type mean values by agreement of the estimated parameters at the 1 mm level for the most part of elevations above 15°, if an identical antenna is used.

Future work includes several refinements that must be made to enable calibration of all GNSS antennas. First, the calibration for BeiDou Navigation Satellite System (BDS) should be feasible in the near future. With the rapid development of BDS, the BDS antenna PCC model is a prerequisite for high accuracy BDS applications, such as, precise point positioning (PPP) and interoperability with GPS and GLONASS and other GNSS systems. Second, our calibration strategies for low elevation need to be further improved as to maintain a consistent precision level with IGS type mean values.
